# Supplementary material for: Antioxidant, Immunostimulatory, and Anticancer Properties of Hydrolyzed Wheat Bran Mediated through Macrophages Stimulation
Source: Int J Mol Sci. 2023 Apr 18;24(8):7436. doi: 10.3390/ijms24087436 (PMC10139194; doi:10.3390/ijms24087436)
Supplement: Supplementary file 1 [file ijms-24-07436-s001.zip › ijms-2338520-supplementary.pdf]

International Journal of
Molecular Sciences

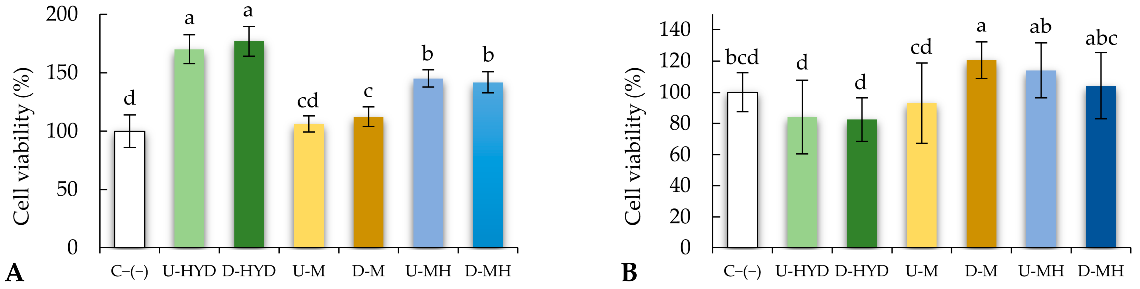

Figure S1. Cell viability of RAW264.7 (**A**) and THP-1 cells (**B**) treated with undigested (U) and digested (D) HYD, M, and MH at 0.5 mg/mL for 24 h. Mean values represented as bars, and standard deviations represented as error bars (n=8). Different lowercase letters show significant differences among experimental groups (one-way ANOVA, post hoc Duncan's test, $p \leq 0.05$). Abbreviations: colon cancer cells treated with growth media without LPS (C−(−)); with growth media with LPS (1 μg/mL) (C+(−)); undigested WB hydrolysate (U-HYD); digested WB hydrolysate (D-HYD); undigested control mousse (U-M); digested control mousse (D-M); undigested mousse with WB hydrolysate (U-MH); and undigested mousse with WB hydrolysate (U-MH).

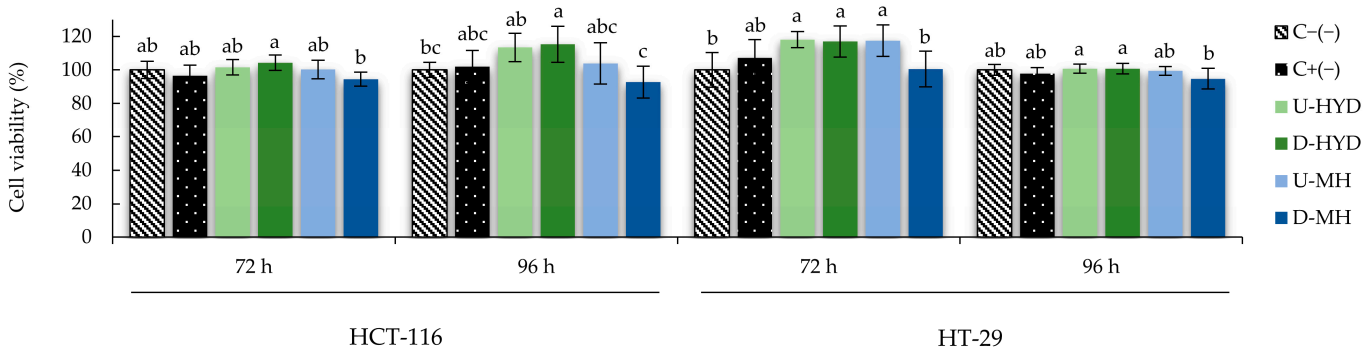

Figure S2. Cell viability of HCT-116 and HT-29 cells exposed to undigested (U) or digested (D) HYD and MH at 0.5 mg/mL for 72 and 96 h. Bars represent means, and error bars represent standard deviations (n = 8). Different lowercase letters show significant differences from each other (one-way ANOVA, post hoc Duncan's test, $p \leq 0.05$). Abbreviations: colon cancer cells treated with growth media without LPS (C−(−)); with growth media with LPS (1 μg/mL) (C+(−)); undigested WB hydrolysate (U-HYD); digested WB hydrolysate (D-HYD); undigested mousse with WB hydrolysate (U-MH); and undigested mousse with WB hydrolysate (U-MH).

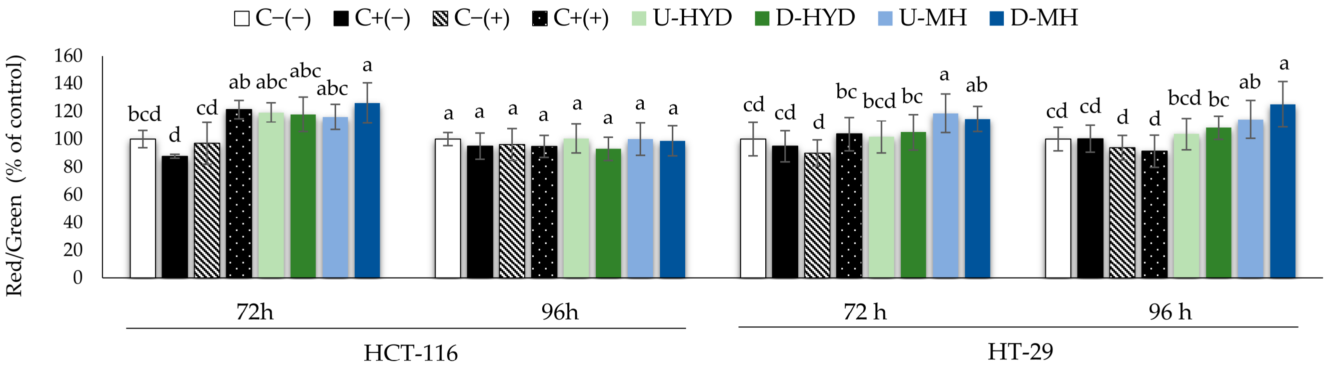

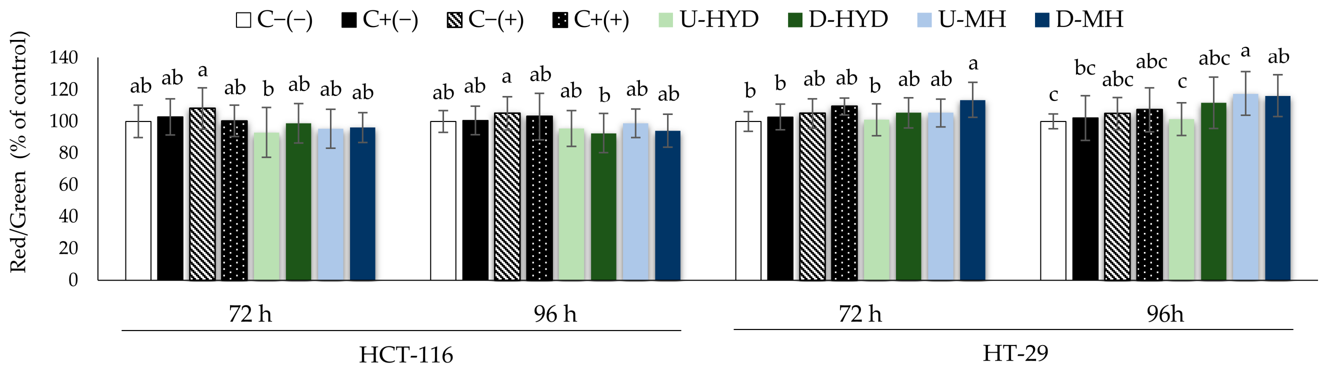

Figure S3. Mitochondrial depolarization using JC-1 die in HCT-116 and HT-29 cells exposed to undigested (U) or digested (D) HYD and MH at 0.5 mg/mL for 72 and 96 h. Numerical data were expressed as % of Red/Green fluorescence[+] cells respect to the control. Bars represent means, and error bars represent standard deviations (n = 8). Different lowercase letters show significant differences from each other (one-way ANOVA, post hoc Duncan's test, $p \leq 0.05$). Abbreviations: colon cancer cells treated with growth media without LPS (C−(−)); with growth media with LPS (1 µg/mL) (C+(−)); with non-stimulated macrophage supernatant (C−(+)); with LPS-stimulated macrophage supernatant (C+(+)); undigested WB hydrolysate (U-HYD); digested WB hydrolysate (D-HYD); undigested mousse with WB hydrolysate (U-MH); and undigested mousse with WB hydrolysate (U-MH).
